# A Case Report on Mid-Dermal Elastolysis: A Distinctive Presentation on the Neck

**DOI:** 10.7759/cureus.45724

**Published:** 2023-09-21

**Authors:** Sabita Aryal, Yaoyu Li, Subodh Bashyal, Liu Ye Qiang, Abdur Rehman

**Affiliations:** 1 Department of Dermatology, Shanghai Skin Disease Hospital, Tongji University, Shanghai, CHN; 2 Department of Endocrinology and Metabolism, Tenth People’s Hospital, Tongji University, Shanghai, CHN; 3 Department of Surgery, Mayo Hospital, Lahore, PAK

**Keywords:** skin wrinkling, elastic tissue disorders, elastophagocytosis, mde, mid-dermal elastolysis

## Abstract

Mid-dermal elastolysis (MDE) is a very rare and acquired skin condition. MDE has a variety of clinical manifestations that can be presented with a reticular erythematous patch with telangiectasis, perifollicular popular protrusions, or finely wrinkled skin. A biopsy is always necessary to rule out other potential elastic fiber disorders.

In this case study, a 33-year-old female with an odd MDE presentation in her neck area is examined. No contributing factors, apart from exposure to sunlight, could be gleaned from the patient's history. The patient didn't benefit from the application of various types of topical agents or any other therapies to lessen the size and advancement of the lesion. In this distinct case, we discuss clinical and histological findings and the treatment plan offered, as well as include a concise review of specific past literature.

## Introduction

A very rare dermatological condition, mid-dermal elastolysis (MDE), is an acquired skin condition first reported by Shelly and Wood in 1977 [1,2]. There have been very few cases reported to date, which makes MDE a very tricky skin disease in terms of knowledge regarding its pathogenesis, etiology, and treatment.

Extracellular matrix components such as fibrillar collagen and elastin contribute significantly to maintaining the structure of the skin [3]. The physical properties of the skin tissues, flexibility, rigidity, and permeability, are determined by the density and arrangement of the collagen and elastin structures [4]. MDE, specifically histopathological, presents with a loss of elastic fibers in the mid-dermis, which leads to diminished elastic recoil and resilience in the affected tissue [5]. Clinical manifestations may include perifollicular papular protrusions, well-defined regions of fine wrinkles, and chronic reticular erythema [6]. We hereby report a rare case of MDE on the right side of the neck.

## Case presentation

**Figure 1:**
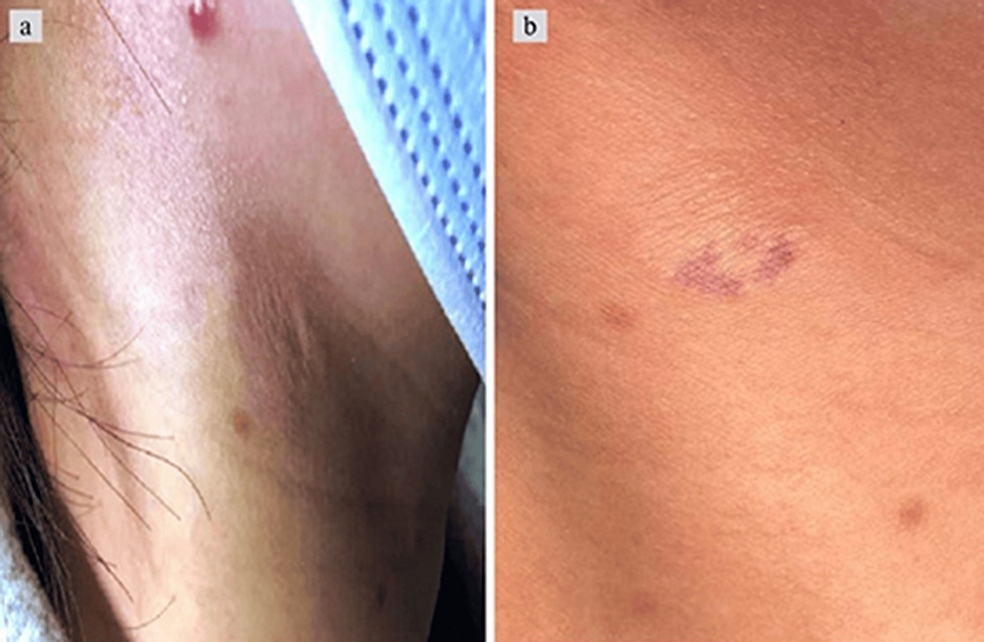
Asymptomatic well-demarcated patch on the right side of the neck (a). A peanut-sized brown patch, measuring 2 to 3 centimeters in diameter (b).

A 33-year-old Chinese female presented at the dermatology clinic with the complaint of skin wrinkling on the right side of her neck. She exhibited well-defined, asymptomatic patches characterized by fine surface wrinkles and superficial skin mobility. Although she had it for the past six years, she first noticed an unusual rash starting to form just over three years ago, eventually leading to the spread of wrinkles around the rash on the right side of the neck (Figure 1a). Upon physical examination, it was discovered that the right side of the neck had a small, peanut-sized brown patch, measuring 2 to 3 centimeters in diameter, with a clearly defined boundary (Figure 1b). The skin in the affected areas appeared normal in color and rebounded after pressure. Her hands, forearms, and lower extremities were free of any lesions, despite areas around her face being sun-damaged. It was noted by her family practitioner four months prior, but she believed to be there for not less than three years.

The patient had been using emollients and occasionally applying topical corticosteroids to alleviate mild itching, but these treatments did not resolve her rash. She was not taking any other medications, including oral contraceptive pills, and had no known allergies. There was no family history of similar skin conditions, and her past medical history was unremarkable. She had not traveled recently but was regularly exposed to sunlight due to her outdoor activities. Dermoscopy examination revealed discrete papule merging with evident linear vessels.

Her blood tests showed no abnormalities in complete blood count, ESR, liver function test, blood glucose, renal function test, serum protein electrophoresis, and autoantibodies, which were not detected. Local excision was done, and a skin biopsy was taken from the right side of the neck, where the skin was wrinkled with a visible brown patch. Histologically, Verhoeff-Van Gieson and H&E stains revealed mid-dermal loss of elastic tissues.

**Figure 2:**
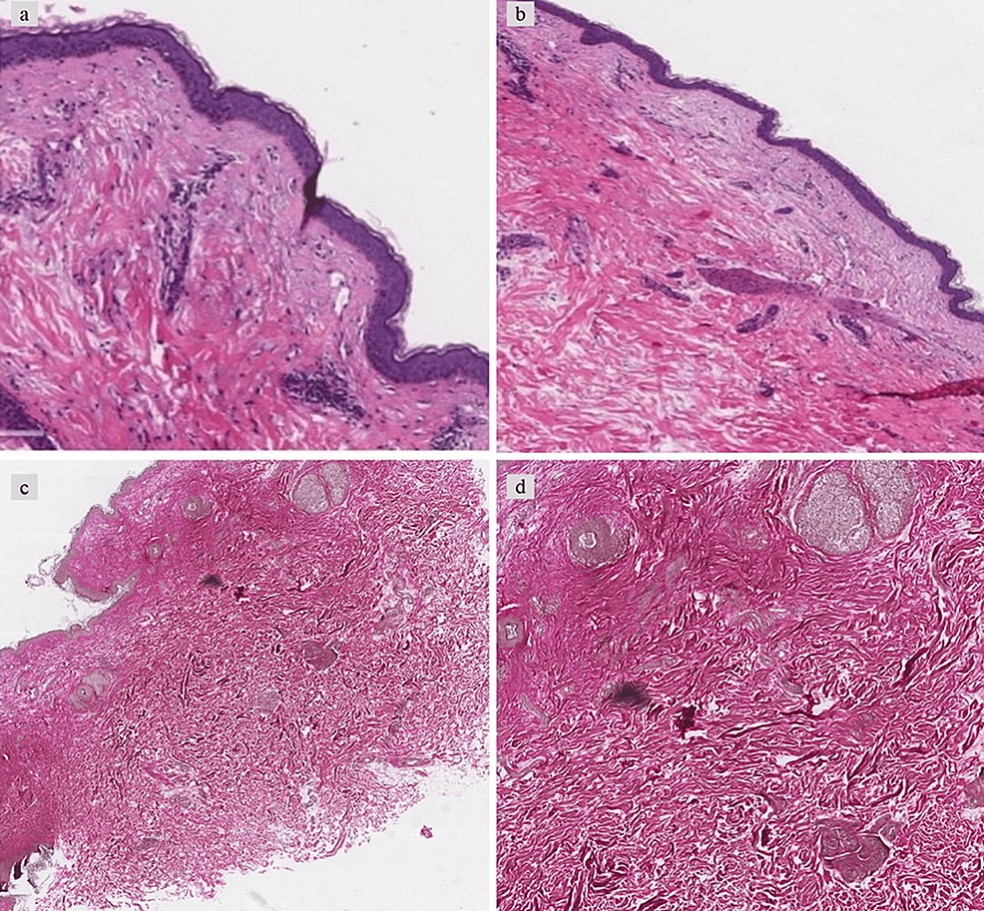
Histopathology shows a moderate perivascular inflammatory cell infiltrate extending to the deep dermis (a and b H&E). Elastin is found to be relatively less in the mid-dermis, compared to the normal area (c and d Verhoeff-Van Gieson stain).

Skin biopsies taken from the affected and surrounding skin of the patient were preserved in formalin and embedded in paraffin for routine H&E staining. The H&E stains revealed a flattened epidermis with a sparsely collagenous middle dermis. The specific loss of elastic tissue was observed in all samples confined within the dermis. However, the papillary layer and the reticular layers, particularly the lower reticular layer of the dermis, didn’t show this infiltration. Additionally, perivascular and peri adnexal inflammatory cell infiltrates were seen extending to the mid-dermis (Figures 2a-2b).

The infiltrate was observed mainly in the areas where elastic tissue was absent and was spread from the superficial reticular dermis to the subcutis. Upon Verhoeff-Van Gieson staining, a visible band-like loss of elastic fibers along with regions of decreased but not a total absence of elastic fibers in the mid-dermis were noted. In MDE, elastin exhibited a significant reduction, while fibrillin remained preserved (Figure 2c-2d). The biopsy of the skin was taken from an area of the lesion, specifically at the edge of the visible wrinkling.

**Figure 3:**
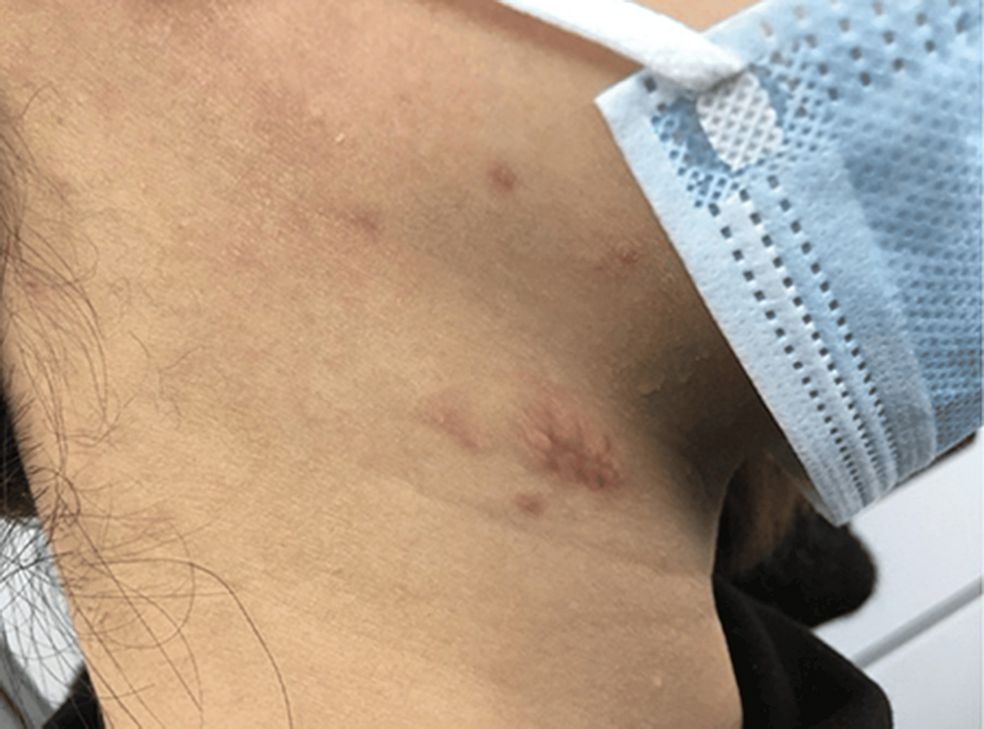
Naked eye observation of the affected area after two months.

Treatment began after the histopathological diagnosis of MDE. Topical application of tacrolimus over the affected area for two months was prescribed. Furthermore, she was instructed to prevent sun exposure over the affected area. Naked eye observation of the lesion after two months of treatment reveals the disappearance of the wrinkling (Figure 3).

## Discussion

MDE is a rare elastic tissue disorder presented with wrinkling of the skin. MDE is known to be an acquired disease that usually affects females in their early 20s to the 60s [6]. It is a dermal inflammation mild in nature and which remains its definitive hallmark. Although inflammatory destruction of the elastic fiber is a major characteristic, the etiology of this condition is still unknown [7]. There aren’t many cases of MDE recorded, and the mild skin abnormalities are still quite unclear and unexposed. These patients frequently don’t show up until a few years after the onset, apparently because the alterations are usually asymptomatic and advance very gently but gradually. The upper extremities, shoulders, back, and trunk are primarily affected by MDE clinically, while the face, soles, and palms are usually spared [8]. However, this case presented a unique feature where the patient exhibited rashes accompanied by skin atrophy in the sternocleidomastoid region. These rashes were preceded by distinct and clearly defined areas of fine wrinkles.

The primary clinical feature observed in our patient was the occurrence of asymptomatic rashes exclusively on the sun-damaged skin of the neck, accompanied by wrinkling while sparing the limbs and face. It is worth noting that elastic fibers possess remarkable regenerative capabilities, allowing successful treatment of these conditions if identified promptly [9].

Three clinical subtypes of MDE have been identified. The most common subtype, Type I, is characterized by asymptomatic, well-demarcated areas of fine wrinkling. Fine wrinkling was the most usual observable clinical alteration of the skin, classified as Type I change by Brenner [6,10]. Type II presents with skin loosening around hair follicles, leading to the development of peri-follicular papules. In a small percentage of instances, the skin around the hair follicles is loose, leading to perifollicular papules with the hair in the center of an umbilication. The coexistence of both lesion types, Type I and Type II, has been observed in several reported cases [11]. Type III presents with reticular erythema. The skin can become diffusely damaged; however, the face is normally unaffected, but the upper body is commonly affected [12].

In the diagnostic process, we considered various other differential diagnosis of elastic tissue disorders such as cutis laxa, anetoderma, pseudoxanthoma elasticum (PXE), and PXE-like papillary dermal elastolysis (PXE-PDE). Cutis laxa is characterized by a generalized loss of elastic fibers throughout the dermis on histopathology. The skin appears loose, saggy, and non-stretchy, similar to that of the elderly. Anetoderma, on the other hand, causes smaller, local flabby, and toneless skin lesions on the trunk and upper extremities, primarily affecting the papillary and mid-reticular dermis. In PXE, yellow papules with a cobblestone appearance are typically found on the lateral side and flexural folds of the neck. For the diagnosis of PXE, a von Kossa stain for calcium is primarily used, where calcium deposits along with clumps of irregularly arranged elastic fibers and basophilic substances are observed.

PXE-PDE clinically resembles PXE but lacks common systemic involvement. Unlike PXE, PXE-PDE does not show calcium depositions on staining. In contrast, MDE affects the mid-dermal layer, and the histopathology of PXE-PDE reveals a band-like loss of elastic fibers in the papillary dermis layer [13].

MDE is a relatively rare dermatological condition, and the management protocol for MDE also varies and includes measures such as sun protection to shield the skin from harmful UV rays, topical retinoids, and topical and systemic steroids, hydroxychloroquine, vitamin E, and colchicine.

However, in this case, the patient’s condition improved with the use of topical tacrolimus and by limiting the affected skin areas from sun exposure. This evidence highlights the importance of accurate diagnosis and appropriate therapy in reducing the manifestations of MDE and enhancing the patient's quality of life.

## Conclusions

MDE remains a rare and poorly understood dermatological condition. This case report contributes to the limited literature by detailing a unique manifestation of MDE in a 33-year-old Chinese female. The patient's condition was successfully managed with topical tacrolimus and sun protection, emphasizing the importance of accurate diagnosis and individualized treatment. The case also highlights the need for further research into MDE's pathogenesis and etiology, as current understanding is limited. In summary, this case report not only enriches the clinical understanding of MDE but also underscores the potential for successful management through targeted therapies. It calls for increased clinical awareness and research to improve early diagnosis and effective treatment, ultimately enhancing patient outcomes.
